# Diagnosis, Treatment and Prognosis of Mesonephric Adenocarcinoma of the Vagina: A Literature Review and a Case Report

**DOI:** 10.3390/jcm12144846

**Published:** 2023-07-23

**Authors:** Federico Ferrari, Andrea Salvatore Omodei, Filippo Alberto Ferrari, Hooman Soleymani Majd, Laura Ardighieri, Salvatore Giovanni Vitale, Antonio Simone Laganà, Stefano Angioni, Giuseppe Ciravolo, Franco Odicino

**Affiliations:** 1 Department of Clinical and Experimental Sciences, University of Brescia, 25123 Brescia, Italy; franco.odicino@unibs.it; 2 Department of Obstetrics and Gynecology, AOUI Verona, University of Verona, 37122 Verona, Italy; ferrarifilippoalberto@gmail.com; 3 Department of Gynaecological Oncology, Churchill Cancer Centre, Oxford University Hospitals NHS Foundation Trust, Oxford OX3 7LE, UK; hooman.soleymanimajd@msd.ox.ac.uk; 4 Department of Pathology, ASST Spedali Civili Brescia, 25123 Brescia, Italy; laura.ardighieri@unibs.it; 5 Division of Gynecology and Obstetrics, Department of Surgical Sciences, University of Cagliari, 09124 Cagliari, Italy; salvatoreg.vitale@unica.it (S.G.V.); sangioni@yahoo.it (S.A.); 6 Unit of Gynecologic Oncology, ARNAS “Civico—Di Cristina—Benfratelli”, Department of Health Promotion, Mother and Child Care, Internal Medicine and Medical Specialties (PROMISE), University of Palermo, 90127 Palermo, Italy; antoniosimone.lagana@unipa.it; 7 Department of Obstetrics and Gynecology, Spedali Civili of Brescia, 25123 Brescia, Italy

**Keywords:** mesonephric adenocarcinoma, vaginal cancer, rare neoplasm, minimally invasive surgery, gynecologic oncology

## Abstract

Background: Mesonephric adenocarcinoma (MA) of the vagina is a rare tumor that arises from mesonephric remnants (Wolffian) in the female genital tract. It is a neoplasm with no significant evidence about its diagnosis, treatment, follow-up and prognosis. Methods: Systematic research of the literature was conducted in Scopus, PubMed/MEDLINE, ScienceDirect and the Cochrane Library, including observational prospective and retrospective studies, case series and case reports. We collected data regarding studies related to diagnosis and treatment options evaluating the following aspects: study design, population, treatment type, rate of surgical complications and fertility outcome. We further included a case report of laparoscopic management of MA with pictorial assays. Results: Thirteen cases of MA of the vagina are available in the literature, including our case report. The median age at diagnosis was 52 years old; the majority of patients reported vaginal bleeding as a symptom (38%); and ultrasound, followed by a magnetic resonance and CT scan were the diagnostic tools most used. In 54% of the cases, a surgical biopsy was performed, and 92% of the patients underwent upfront surgery with an open access or vaginal resection except one case fully managed by minimally invasive surgery. Most of the patients (68%) received adjuvant treatment with chemotherapy or radiotherapy or a combination of them. The mean follow-up period was 6 years. Conclusions: Despite the rarity of this cancer and bizarre location, a minimally invasive approach seems feasible after multidisciplinary evaluation. According to the rarity of this tumor, any future case and follow-up data must be reported in the literature in order to enlarge the knowledge about it.

## 1. Introduction

Mesonephric adenocarcinoma of the vagina is a rare neoplasm that arises from the mesonephric remnants or hyperplasia in the female genital tract. The Wolffian (mesonephric) ducts in male individuals form the seminal vesicles, epididymis, vas deferens and efferent ducts of the testis. Instead, in females, they eventually regress. Although the remnants are a common finding, up to one-third of women’s lower genital tract are mainly found only in the form of isolated groups of cells in the broad ligament, in the lateral wall of the cervix or in the vagina. Their presence, even in the form of a benign pathology such as the Gartner duct cyst, is not considered a risk factor to develop cancer. MA risk factors and etiology are still unclear; mesonephric hyperplasia is considered a premalignant lesion; however, its evolution into cancer is rare, and complete excision seems to significantly reduce the risk [1]. MAs are mainly located in the cervix and vagina and, less likely, in the upper genital tract, where mesonephric-like adenocarcinomas (MLAs) arise more frequently from mesonephric transdifferentiation of Mullerian carcinomas [2].

Primary vaginal carcinoma is uncommon, accounting for only 1–2% of all gynecological malignancies, and MA represents less than 0.1% of vaginal cancers [1]. Few cases have been reported in the literature and uniformity in evidence was labored by a change in nomenclature: originally the clear cell adenocarcinoma (CCAs) of Mullerian origin was called mesonephroma because of its histological similarities to CCAs of the kidney (i.e., mesonephros) creating possible misleading classification [3]. This neoplasm is considered a real challenge for clinicians and pathologists that are referred to for vaginal cancers and they must be aware of this entity. The lack of specific guidelines supporting MAs’ management is related to the rarity of the disease, and to date, no report on laparoscopic management has been reported in the literature [3].

Even though the treatment of vaginal cancer is based upon retrospective studies, no RCTs have been performed to define the gold standard of treatment. Management must consider cancer staging, and the patient’s age, sexual activity and childbearing desire, aiming to treat the tumor with the lowest impact on the patient’s quality of life. Radiation therapy is the most frequent choice in advanced stages and represents the cornerstone in vaginal cancer therapy in order to guarantee organ preservation; concurrent chemotherapy with cisplatin or 5-FU is also considered in the literature. Surgery is performed in stage I tumors limited to the mucosa of the vagina: radical hysterectomy and additional pelvic lymphadenectomy is more commonly proposed for upper vaginal cancers, while tumor resection and additional groin lymphadenectomy is the choice for lower vaginal cancers [4].

Based on this scenario, the present study aims to provide additional evidence and summarize the available evidence in the literature concerning the diagnosis, treatment and prognosis of MA of the vagina, further reporting a unique case of laparoscopic management of the disease.

## 2. Materials and Methods

### 2.1. Study Design and Search Strategy

We conducted a systematic review of the literature by performing a literature search in the electronic database Scopus, PubMed/MEDLINE and ScienceDirect from the database inception to January 2023. A combination of keywords was used as follows: “Mesonephric carcinoma” OR “Mesonephric adenocarcinoma” AND “vagina”.

### 2.2. Inclusion Criteria

The study aimed to ask the following PICOS items. Population: women with MA of the vagina; intervention: surgical, radiological and medical management for MA of the vagina; comparators: no comparators; outcomes: to summarize the available evidence of the MA vaginal cases and to identify the most frequently diagnostical and therapeutic approaches; study design: observational studies, case reports, case series and reviews were included; language: no restriction.

### 2.3. Study Selection and Data Extraction

One author (ASO) independently screened titles and abstracts from the studies in the search result and extracted the following data: study features (authors, year of publication, number of cases), population (age at diagnosis, previous gynecological surgery), characteristics of the disease (clinical presentation, radiological features, size, histology), management of the disease (diagnostical features, preoperative investigation, type of treatment and type of surgical approach), follow-up data. One other author double checked data extraction and provided clinical and scientific insights (FAF).

### 2.4. Data Synthesis

A standardized form was used to extract data from included studies. The data extracted were evaluated by analyzing the following features: age of the patient, anamnesis of previous surgery, clinical manifestations, size of the tumor, diagnostic imaging techniques, availability of tumor markers, presurgical biopsy, additional diagnostic procedures, surgical approach, surgical treatment, peritoneal washing, neoadjuvant or adjuvant treatment, FIGO staging, presence of local invasion, presence of lymph node involvement, metastatic localizations and prognostic data from follow-up screening.

## 3. Results

### 3.1. Case Report

A 38-year-old woman was referred to our gynecological ultrasound service for the follow-up of a known ovarian cyst. Her history was silent for previous abdominal or gynecological surgery; she had vaginally delivered twice at term and had experienced retinal detachment 4 years before. Familiar anamnesis was silent for oncological diseases. The ultrasound scan (US) described a probable mass of ovarian origin of 42 × 43 × 45 mm, with mixed structure, color-score 1, suspected as the first hypothesis to be a dermoid. Nonetheless, the possible vaginal origin could not be excluded and the patient underwent an MRI scan. It revealed a complex cyst of 51 × 49 × 42 mm localized at the upper third of the vagina, hyperintense at T1w sequences and with strong enhancement after Gadovist™ injection with multiple solid components, suspected for malignancy. The radiological evaluation did not show any lymphatic involvement or distant localization. The tumor markers were negative except for a slight increase in CA 125 (43 UI/mL).

**Figure 1:**
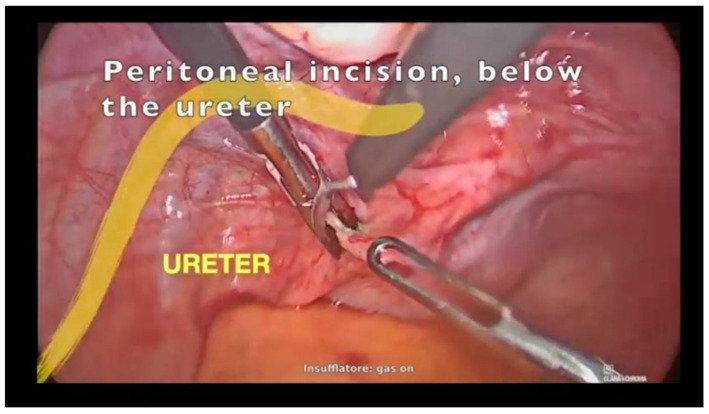
Incision of the peritoneum below the ureter.

The patient was scheduled for minimally invasive surgery. After the induction of the pneumoperitoneum and entrance in the abdomen, the bulging neoformation was transperitoneally identified below the ureter (Figure 1) and behind the caudal limit of the left uterosacral ligament.

**Figure 2:**
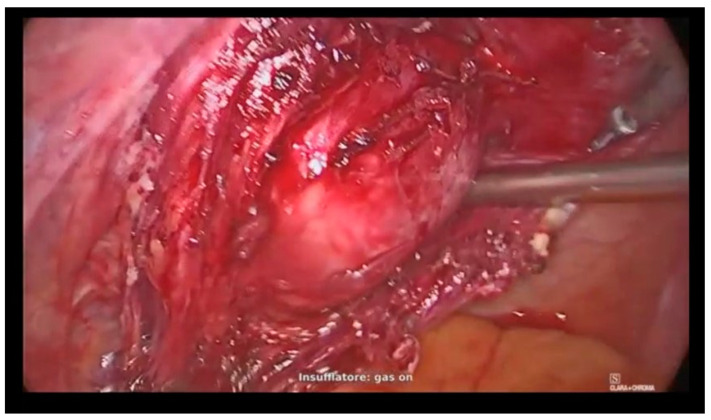
Identification of a cleavage plane up to the vaginal limit.

Medial approach to the retroperitoneum and development of the Okabayashi space was performed until the identification of a cleavage plane up to the vaginal limit (Figure 2).

**Figure 3:**
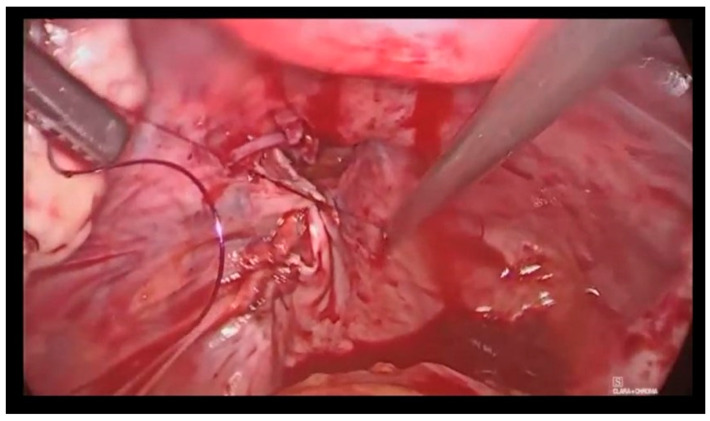
Intracorporeal suture of the peritoneum.

Subsequently, we performed the incision of the vaginal wall, preventing the loss of pneumoperitoneum with the inflation of a pneumo-occluder in the vagina. After the identification of the ventral and dorsal limits of the cyst, we performed an en-block excision of the aforementioned formation and the contiguous vaginal wall and we removed the specimen in an endobag through the vagina. Finally, transvaginal closure of the wall defect with a continuous suture and intracorporeal suture of the peritoneum were performed (Figure 3).

Intraoperative histological evaluation confirmed the malignant nature of the lesion with no extracapsular invasion. A sub-centimeter suspected endometriosis peritoneal localization was removed. A bilateral salpingectomy was completed on the patient’s request. The post-operative course was regular and the patient was discharged on the second post-operative day.

**Figure 4:**
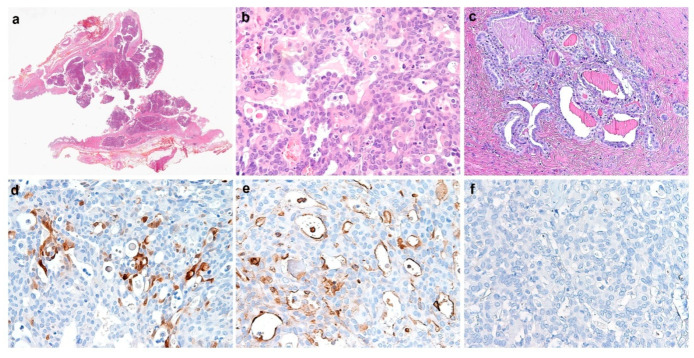
(**a**) Intracystic mesonephric carcinoma of the vagina, with no evidence of extracapsular invasion (H&E, whole tumor section); (**b**) mesonephric carcinoma of the vagina with tubular pattern, showing densely eosinophilic intraluminal secretions (H&E, 20×) and (**c**) mesonephric remnants detected in vaginal wall (H&E, 10×). (**d**–**f**) Immunohistochemical profile of mesonephric carcinoma, namely tumoral cells, were positive for calretinin ((**d**), 20×)) and CD10 ((**e**), 20×)), while negative for progesterone receptor ((**f**), 20×)).

The final histological report provided the diagnosis of intracystic MA of the vagina with no extracapsular invasion, arising from mesonephric remnants in the context of a normal vaginal epithelium; the fallopian tubes were normal, peritoneal washing was negative for malignancy and the peritoneal biopsy confirmed the endometriosis. The immunohistological staining performed on the tumoral tissue was positive for pancytokeratin, PAX8, calretinin, CD10 and PAX2, while it was negative for estrogen receptor (ER), progesterone receptor (PR), TTF-1, GATA-3, vimentin and p53. The clinical case was discussed by a multidisciplinary team including gynecologic surgeons and oncologists, radiotherapists, pathologists and radiologists (Figure 4).

According to the intracystic localization of the tumor (FIGO stage I) [4], no further treatment was suggested and the patient was indicated to clinical and radiological follow-up. The patient was free from disease 15 months after surgery. MRI and US evaluation during the follow-up demonstrated no evidence of recurrence. She underwent diagnostic hysteroscopy one year after surgery because of abnormal uterine bleeding and the biopsy revealed an endometrial polyp without atypical features.

### 3.2. Systematic Review of the Literature

**Figure 5:**
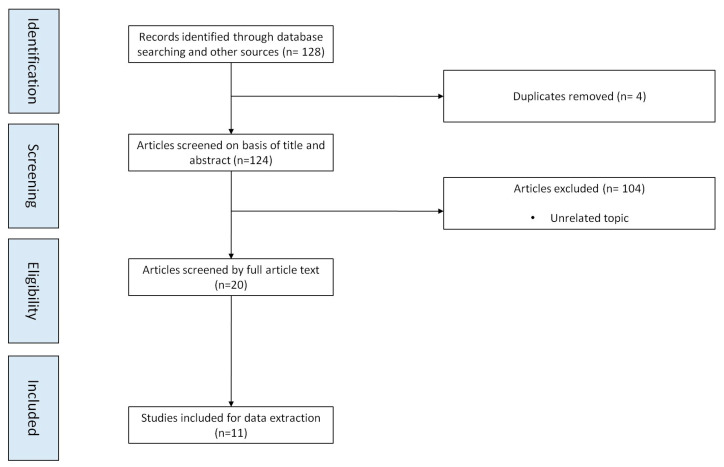
Flow diagram of studies’ selection.

The search strategy retrieved 128 items. After duplicates’ removal (n = 4) and screening of all the manuscript titles and available abstracts (n = 103), 20 studies were assessed for full text evaluation. Finally, 11 of those were elegized for data extraction. The flowchart of study selection is shown in Figure 5.

#### 3.2.1. Historical Findings

In the literature, the first online available case report of MA of the vagina dates back to 1955, when Wahlèn T. and Gynning I. described a case of a 38-year-old woman affected by Gartner’s duct carcinoma treated with neoadjuvant radiotherapy and radical surgery [5]. Later, Grunberger W et al. in 1977 [6] and Shevchuck MM et al. in 1979 [7] described similar tumors pointing out that the so-called mesonephroma was different in origin (Mullerian CCA) rather than the MA arising from the Gartner duct (Wolffian). There were also reported cases of vaginal MA published in the early 1980s in Japanese literature: Nemoto et al. in 1983 [8] and Tanigawa et al. in 1985 [9]. Although the full texts of these papers are not available, this review summarizes the main findings from 2004 up to date for a better availability of information and the possibility of comparing the outcomes of this review.

#### 3.2.2. Characteristics of Patients, Clinical Manifestations and Diagnostical Features

Together with our reported patient and the results of the literature review, a total of 13 cases of MAs of the vagina were found [2,10,11,12,13,14,15,16,17,18,19] (Table 1). The median age at diagnosis was 52 (from 22 to 63). The clinical manifestation at diagnosis varied: the most were referred to a gynecologist for vaginal bleeding (5/13—38%), including post-coital bleeding; two patients manifested vaginal swelling or discomfort; two had pelvic pain or dyspareunia; one underwent an anomalous Papanicolaou smear, one was referred to the gynecologist for leiomyomas follow-up; one had a US scan for a suspected ovarian cyst; one had a vaginal polyp at speculum examination; and one had urinary urgency. Five patients had a past history of gynecological surgery: one patient underwent a transabdominal hysterectomy (TAH) and bilateral salpingo-oophorectomy (BSO), one patient a TAH alone for myomas, one patient a supracervical hysterectomy (SCH), one patient a vaginal hysterectomy (VH) for leiomyomatosis and one patient had undergone two previous cesarean sections (CSs). None of them had been reported to have a previous oncological history.

The mean size of the tumor at diagnosis was 43 mm (from 10 to 140 mm). The tumor markers (TM) were reported in only four cases (30.8%): two patients had negative TM; and two patients had elevated carbohydrate antigen 125 (CA125). Pelvic US was the most frequent radiodiagnostic method used to evaluate the pelvic structures. Five patients underwent magnetic resonance imaging (MRI) and three patients were evaluated by computed tomography (CT). Additional diagnostic methods were required in case of suspicion of local invasion and two patients had presurgical cystoscopy for urethra and bladder invasion assessment. To preoperatively determine the nature of the lesion, a punch biopsy of the tumor was performed in seven cases. Our case is the only one with an intraoperative histological confirmation of malignancy.

#### 3.2.3. Therapeutical Management and Prognosis

The findings are summarized in Table 2. Surgery was the primary treatment in most of the cases (12/13; 92%): laparotomy (LPT) and vaginal surgery were performed in 46% and 31%, respectively. In one case, the type of surgery was not specified. To our knowledge, the proposed case is the first case managed with a minimally invasive approach. Six patients received radical tumorectomy (ResTu) (one of them with additional BS, another with additional BSO), while one patient was treated with R1 ResTu to avoid further surgical comorbidities. Two patients underwent TAH and BSO (one of them with additional colpectomy), and one pelvic exenteration with ileal conduit was performed. Laparotomic bilateral pelvic lymphadenectomy (BPLND) was proposed in three patients, and in one case, lymph node involvement was confirmed. Peritoneal washing for cytologic examination was performed in two cases, one of which resulted as positive. Local invasion was present in six cases and was reported as negative in four patients. No cases had distant metastasis.

According to the 2009 Figo staging for vaginal cancer [4], five patients were classified Stage I, four patients were Stage II, three were stage III, and in one case, staging was not reported. Additional treatment was considered in eight patients with local invasion or with locoregional metastasis. One patient received neoadjuvant brachytherapy (BT) before surgery, while seven patients received adjuvant treatment after surgery: three cases of chemotherapy (CHT), external beam radiotherapy (EBRT) and BT; two patients were treated with BT alone, one patient with CHT plus EBRT and one patient with CHT alone.

Follow-up data are available in 11 patients out of 13, all of them were free from disease to the date of the case report without any recurrence. The mean follow-up period at the report time was 6 years (from 6 weeks to 11 years). No recurrence was reported. One patient who had MA and right renal agenesis was free from disease 11 years after EBRT plus CHT with vaginal stenosis and hydroureteronephrosis.

## 4. Discussion

MA of the vagina is a rare tumor that represents a diagnostic challenge for gynecologists and pathologists. Little is known about its biologic, diagnostic, therapeutic and prognostic features. Few reports are present in the literature, making it difficult to understand the tumor’s behavior. Because of the paucity of data, no specific guidelines are available. Diagnosis, staging and treatment of MA of the vagina should follow the guidelines for vaginal cancer [19]. Vaginal MA may occur as a polypoid mass arising from the vaginal wall and protruding into the lumen [11], or a cyst bulging into the peritoneal cavity, as in the reported case from our Department. The literature review showed that vaginal bleeding is the most frequent symptom at diagnosis.

Differential diagnosis included vaginal cysts (in particular Gartner duct cysts that arise from Wolffian remnants such as MAs), vaginal polyps, urethral diverticulum, pelvic floor dysfunction and vaginal wall prolapse, Bartholin’s and Skene’s gland cyst or abscess, vaginal intraepithelial neoplasia, vaginal adenosis, mesonephric hyperplasia, endometriosis and other vaginal malignancies such as squamous cell carcinoma, adenocarcinoma, clear cell carcinoma, sarcoma botryoids and vaginal melanoma [1,4]. The diagnostic work-up should include US and MRI for local assessment, and eventually CT and positron emission tomography (PET)-CT for staging and evaluation of distant localization. To adequately plan the surgical strategy, additional investigations such as cystoscopy or proctoscopy in the case of a suspicion of bladder or rectum invasion can be considered [19]. Tumor markers do not seem to be useful at diagnosis, although they have to be performed to exclude other malignancies. Pretreatment biopsy is recommended in order to identify histotype and grading, and to perform immunostaining. PAX8, CD10 and HNF1b are commonly positive, while GATA-3, TTF-1, calretinin and inhibin are less frequently positive; CEA, ER and PR are usually negative. High-risk human papilloma virus seems to not represent a risk factor for vaginal MAs and p16 immunostaining is generally weak or negative [1]. A study by Lin et al. [20] analyzed the molecular profiling of MAs and MLAs showing that KRAS seems to be the main mutated oncogene that drives the carcinogenesis (90% of cases), together with ARID1A, PI3CA, TNNB1, TP53, MLL2 and CDKN2A. Consistent with other vaginal cancers, the aim of the treatment is to guarantee an oncological adequacy, and preserve the reproductive potential and sexual function.

No randomized controlled trials have been performed, and the actual guidelines are based on retrospective studies on other histotypes of vaginal cancer; MAs’ prognosis in unknown because of a lack of evidence.

Complete resection of the mass with one cm free margins was demonstrated to be a feasible and safe option for lower genital disease at an early stage. In the case of preoperative suspicions, pelvic or groin lymph-nodal assessment could be performed according to the location of the tumor mass. Upper vaginal disease must be carefully evaluated and, in our experience, it was feasible to avoid a hysterectomy because of the intracystic localization of the tumor and the childbearing desire of the patient. In our case, it was possible to proceed with a minimally invasive approach, given the localization of the disease and based on strong experience in laparoscopic complex surgery in the field of gynecological oncology [21]. Even though guidelines suggest radical hysterectomy and vaginectomy with 1 cm margins together with PLND, we discussed with the patient the management of the disease given its rarity and we decided for a close follow-up. Advanced disease must be treated radically, and additional EBRT + BT with concurrent CHT with cisplatin or 5-FU may be considered the primary choices. Both adjuvant and neoadjuvant treatment have been reported in literature [4].

Actually, there is a lack of evidence for the feasibility and effectiveness of minimally invasive treatment and fertility-sparing surgery for vaginal cancer: two case reports found in the literature deal with laparoscopy surgical treatment for vaginal intraepithelial neoplasia in patients that had both undergone previous hysterectomy and had the lesion on the vaginal cuff [22,23].

Follow-up is established according to the guidelines for vaginal cancer; particular attention may be given to those patients that present wolffian remnant lesions such as Gartner duct cysts or mesonephric hyperplasia that may represent a risk factor for recurrence. In the literature, the case of a clear cell carcinoma arising from Gartner duct cyst has been reported [24].

Knowledge is growing about sexual toxicity in women that undergo pelvic radiotherapy, with most of the data deriving from endometrial and cervical cancer treatment [25], but it can be tailored to those patients with vaginal cancer that are treated with EBRT and BT. Vaginal dilatator and topical therapy can reduce or even prevent vaginal stenosis and synechiae.

## 5. Conclusions

MAs of the vagina, as with other histotypes of vaginal cancers, have to be centralized to a specialized gynecologic oncology center in order to achieve a correct diagnosis and guarantee multidisciplinary management and adequate treatment. In our case report, despite the rarity of this cancer and bizarre location, a minimally invasive and fertility sparing approach with conservative aims seems feasible, and to date, it has guaranteed the patient being free from disease.

Prognosis of MA is unknown due to a lack of evidence; it may be useful for every new case of MA and recurrence of MA to be reported in the scientific literature because further data are necessary to understand the nature and behavior of this tumor to provide specific guidelines in the future.

## Figures and Tables

**Table 1:** Characteristic of patients, clinical manifestations and diagnostical features.

Author, Year	Baguè, 2004	Baguè, 2004	Ersahin, 2005	Bifulco, 2008	Mueller, 2016	Roma, 2014	Amal, 2015	Plesinac, 2017	Shoeir, 2018	Xie, 2021	Lee, 2022	Kumar, 2022	Case Report
Previous Surgery	-	-	VH	TAH, BSO	-	SCH	-	-	2 CS	-	-	TAH	-
Cystoscopy	-	-	-	-	yes	-	-	-	yes	-	-	-	-
Presurgical Biopsy	MH	-	ADC	-	Hyperplasia	Mullerian tumor	ADC	MA	-	-	ADC	-	IOA: ADC
CA 125	-	-	-	pos	-	-	-	-	-	neg	neg	-	pos
Imaging	-	-	-	US, CT	MRI	US, CT	MRI	CT	US, MRI	-	MRI	-	US, MRI
Size (cm)	4	-	1	14 × 7 × 6	2.5 × 1.8	5 × 2.5 × 0.5	4	-	3.1 × 2.7 × 2.9	-	2.5	2 × 1.5 × 1	5.1 × 4.2 × 4.9
Symptoms	Leiomyomas	Dyspareunia	Polyp, + PAP smear	Pelvic pain, Pruritus vulvae	Post-coital vaginal bleeding	Vaginal bleeding	Vaginal bleeding	-	Vaginal swelling, urinary urgency	Vaginal discomfort	Vaginal bleeding	Vaginal bleeding	Ovaric cyst
Age	54	38	55	58	54	58	50	22	63	31	52	40	39

IM: imaging, CA125: carbohydrate antigen 125, US: ultrasonography, CT: computed tomography, MRI: magnetic resonance imaging, MH: mesonephric hyperplasia, ADC: adenocarcinoma, MA: mesonephric adenocarcinoma, IOA: intraoperative assessment, VH: vaginal hysterectomy, TAH: transabdominal hysterectomy, BSO: bilateral salpingo-oophorectomy, SCH: supracervical hysterectomy, CS: cesarean section.

**Table 2:** Therapeutical management and prognosis.

Authors, Year	Baguè, 2004	Baguè, 2004	Ersahin, 2005	Bifulco, 2008	Mueller, 2016	Roma, 2014	Amal, 2015	Plesinac, 2017	Shoeir, 2018	Xie, 2021	Lee, 2022	Kumar, 2022	Case Report
Follow-up	8y7m—PFS	-	3y—PFS	1y—PFS	4y—PFS	1m—PFS	-	11y—PFS	2m—PFS	7y6m—PFS	10m—PFS	-	1y3m—PFS
Adjuvant Treatment	-	-	BT, EBRT, CHT	-	BT, EBRT, CHT	-	BT (NeoAdj.)	EBRT, CHT	BT	CHT	BT, EBRT, CHT	BT	-
N	-	-	pos	neg	neg	neg	-	-	neg	neg	neg	-	Neg
Local Invasion	pos	-	pos	neg	pos	pos	pos	-	neg	neg	pos	-	Neg
FIGO Stage	II	-	III	I	II	II	III	III	I	I	II	I	I
Washing	-	-	pos	-	-	-	-	-	-	-	-	-	neg
Surgery	TAH, BSO, CPT	ResTu	BSO, CPT, PLND	ResTu, PLND	ResTu (R1)	Pelvis exenteration, Ileal conduit	unknown	-	ResTu (+ spilling)	TAH, BSO	ResTu, BSO, PLND	ResTu	ResTu, BS
Surgical Approach	LPT	VS	LPT	LPT	VS	LPT	unknown	-	VS	LPT	LPT	VS	LPS
Age	54	38	55	58	54	58	50	22	63	31	52	40	39

LPT: laparotomy, VS: vaginal surgery, LPS: laparoscopy, TAH: transabdominal hysterectomy, BSO: bilateral salpingo-oophorectomy, BS: bilateral salpingectomy, CPT: colpectomy, PLND: pelvic lymph node dissection, ResTu: tumorectomy, N: lymph-nodal invasion, BT: brachytherapy, EBRT: external beam radiotherapy, CHT: chemotherapy, PFS: progression-free survival.

## Data Availability

No new data were created or analyzed in this study. Data sharing is not applicable to this article.
